# Local Water-Filling Algorithm for Shadow Detection and Removal of Document Images

**DOI:** 10.3390/s20236929

**Published:** 2020-12-04

**Authors:** Bingshu Wang, C. L. Philip Chen

**Affiliations:** 1 School of Software, Taicang Campus, Northwestern Polytechnical University, Suzhou 215400, China; wangbingshu@nwpu.edu.cn; 2 School of Computer Science and Engineering, South China University of Technology, Guangzhou 510006, China; 3 Faculty of Science and Technology, University of Macau, Macau 999078, China

**Keywords:** local water-filling algorithm, topographic surface, shading map, document image, penumbra removal

## Abstract

Shadow detection and removal is an important task for digitized document applications. It is hard for many methods to distinguish shadow from printed text due to the high darkness similarity. In this paper, we propose a local water-filling method to remove shadows by mapping a document image into a structure of topographic surface. Firstly, we design a local water-filling approach including a flooding and effusing process to estimate the shading map, which can be used to detect umbra and penumbra. Then, the umbra is enhanced using Retinex Theory. For penumbra, we propose a binarized water-filling strategy to correct illumination distortions. Moreover, we build up a dataset called optical shadow removal (OSR dataset), which includes hundreds of shadow images. Experiments performed on OSR dataset show that our method achieves an average ErrorRatio of 0.685 with a computation time of 0.265 s to process an image size of 960×544 pixels on a desktop. The proposed method can remove the shading artifacts and outperform some state-of-the-art methods, especially for the removal of shadow boundaries.

## 1. Introduction

Optical shadows appear out of nowhere in the images captured from camera sensors [1,2,3,4]. They are generated when light sources are occluded by static or moving objects [5,6,7,8]. In most cases, shadows are regarded to be useless and need to be removed from images. One of the most used engineering applications is to remove optical shadows from document images.

With the increasing use and popularization of smart phones, people are more likely to use them as a mainstream document capture device rather than a conventional scanner. As a result, many document images are captured under various situations and conditions such as indoor and outdoor. Since the occlusion of illumination sources in environments is inevitable, shadows usually appear in the document images [1,9] with different types: weak, moderate, strong or nonuniform [10,11].

When document images are cast by shadows, the occluded regions become darker than before. It is observed that the text is always printed in black on the documents. Specifically, when the darkness of the shadows is similar to that of text, it will generate poor-quality text [9,11]. The shadows may make the perception of documents uncomfortable to the human eye and cause the degradation of text in documents or notes, which will result in difficulties for text binarization and recognition [12,13]. Therefore, removing shadows from document images not only helps generate clear and easy-to-read text [14], but also makes document binarization [15,16] and recognition tasks [17,18,19] possible.

Over the past decade, shadow removal is playing a growing role in digitized document application and attracting the attention of many researchers. Bradley et al. [20] proposed an adaptive threshold technique for binarization utilizing the integral image that is calculated from the input image. It is sensitive to a slight illumination change but it cannot remove the boundaries of strong shadows. Bako et al. [14] came up with a strategy that estimates local text and background color in a block. They removed shadows by generating a global reference and a shadow map. Shah et al. [21] considered shadow removal as an estimation problem of shading and reflectance components of the input image. An iterative procedure was explored to handle hard shadows. However, the large number of iteratations required too many calculations.

The method proposed by Kligler et al. [11] developed a technique of 3D point cloud transformation for visibility detection. It aims to generate a new representation of an image that can be used in common image processing algorithms such as document binarization [22,23] and shadow removal [14]. However, the transformation process requires huge computational power. The approach proposed by Jung et al. [24] explored a water-filling method to rectify the illumination of digitized documents by converting the input image into a topographic surface. It is implemented based on the YCbCr color space and only takes the luminance component into account. It achieves good performance on weak or medium shadows. However, this method tends to produce degraded color results for scenes with strong shadows.

On one hand, shadows need to be removed. On the other hand, obvious color artifacts should be avoided after shadow removal. Zhang et al. proposed a prior-based [25] method and learning-based [26] method for removing color artifacts. Barron et al. proposed a fast fourier color constancy method [27] and a convolutional color constancy method [28] to recover a white-balanced image and make the image natural-looking. These methods are expected to provide potential means to correct the non-uniform illumination and color artifacts. In addition, there are other methods proposed to detect shadows [29,30,31,32], and remove shadows from document images or natural images [33,34,35,36,37,38], which is expected to benefit many text detection and recognition approaches reviewed in [39].

Physically, shadows can be divided into two parts: umbra and penumbra [40]. For weak or medium shadows, the umbra and penumbra have fuzzy boundaries and can both be handled by the methods mentioned above. However, for strong shadows, these methods face challenges. There are two possible reasons for this. On one hand, shadow strength is difficult to estimate for shadow regions. On the other hand, many shadow points belong to shadow boundaries and they are very similar with surrounding texts.To remove these shadows, some works have been completed. Some datasets have been created for research on document shadow removal, for example, the Adobe [14] and HS datasets [21]. However, only a few images in these datasets have strong shadows. Therefore, it is necessary to build up a dataset that includes more images with strong shadows.

**Figure 1:**
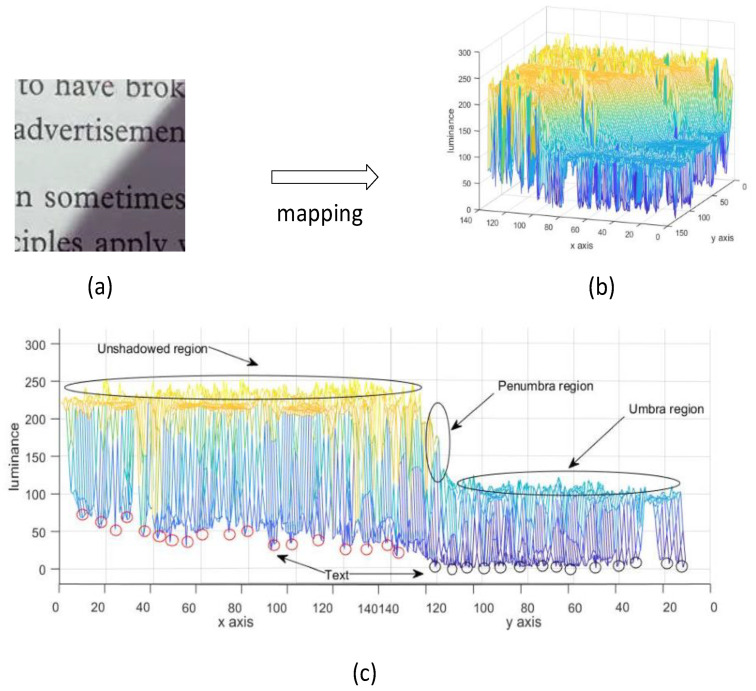
The mapping illustration from one document image with shadows to its topographic structure. (**a**) one document image with shadows, (**b**) the visual topographic structure of image (**a**), (**c**) the clarification of mapping process from image (**a**) to image (**b**): the unshadowed region can be regarded as plateau, umbra as catchment basin, the penumbra as ridge between plateau and basin, and text as the lowest points.

Our motivation is to explore a means to remove shadows from document images. In this paper, we solve the problem by mapping an image into a topographic surface, i.e., unshadowed region can be regarded as plateau, umbra as catchment basin, and the penumbra as ridge between plateau and basin, which is shown in Figure 1. This paper devises a design to obtain a shading map using local water-filling (LWF), which helps to estimate shadow strength. To remove shadow boundaries, this paper proposes a local binarized water-filling (LBWF) algorithm to correct illumination distortions of document images. Moreover, we create a dataset that includes many images with strong shadows.

The contributions of this paper are as follows:

(1) This paper designs a local water-filling approach to estimate a shading map using a stimulation of flooding and effusing processes (Section 2.1). This strategy is able to produce an effective map that indicates the shading distribution in a document image.

(2) This paper develops a local binarized water-filling algorithm for penumbra removal (Section 2.4). This provides an effective means to remove strong shadow boundaries, which is a difficult problem for many methods due to the high similarity between penumbra and text.

(3) We create a dataset called OSR for shadow removal in document images, including the controlled illumination environment and natural scenes. Specially, the dataset contains some typical scenes with strong shadows (Section 3.1).

(4) The proposed method’s efficiency is superior to some state-of-the-art approaches as the experiments are conducted on an image with a size of 960×544 pixels.

The paper is organized as follows. Section 2 presents the proposed method. Section 3 gives the experimental results and analysis. Section 4 concludes this work.

## 2. The Proposed Method

**Figure 2:**
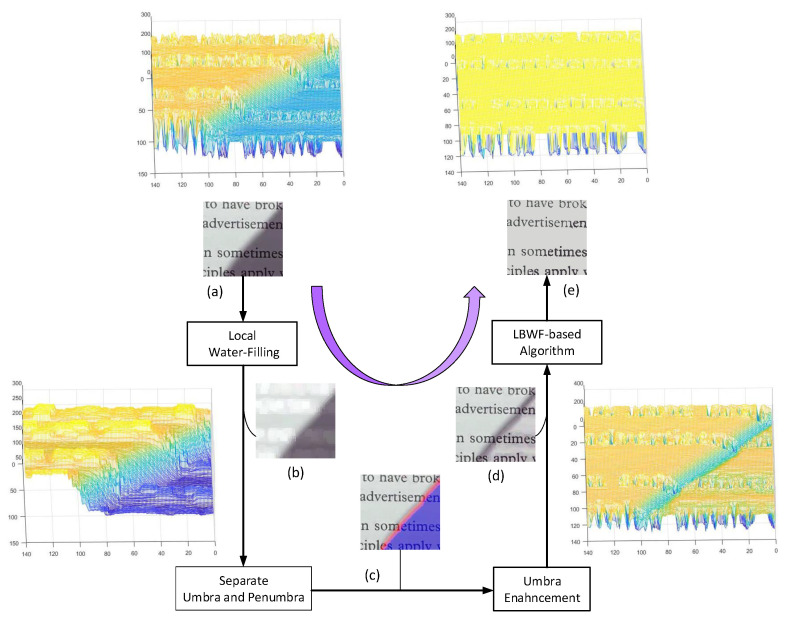
The flowchart of optical shadows removal. (**a**) input image, (**b**) shading map, (**c**) the red represents umbra and the purple represents penumbra, (**d**) the image after umbra enhancement, (**e**) the output image without shadows.

The flowchart of the proposed method is presented in Figure 2. Firstly, the proposed local water-filling (LWF) algorithm receives an input image with shadows and generates a shading map (see Figure 2b) which represents local background colors of the input image. The shading map can be used to detect umbra (the red) and penumbra (the purple) (as shown in Figure 2c). Then, the umbra can be relighted according to Retinex theory (Figure 2d). Finally, a local binarized water-filling-based (LBWF-based) algorithm was designed to remove the shadow boundaries and produce an unshadowed image (Figure 2e). Notably, Figure 2 shows the topographic structures of the image (a), (b), (d) and (e), indicating how the topographic surface changes.

### 2.1. Local Water-Filling Algorithm

In this section, we report a design to estimate a shading map of the input image using a local water-filling algorithm. It mainly includes two parts: a flooding and effusing part. This paper stimulates this process by solving three core problems: where does the “water” come from; where does the “water” flow out; how is the “water” stored. The proposed algorithm is modeled by figurative flowing of “water”. Therefore, some variables need to be defined first before modeling our method.

**Figure 3:**
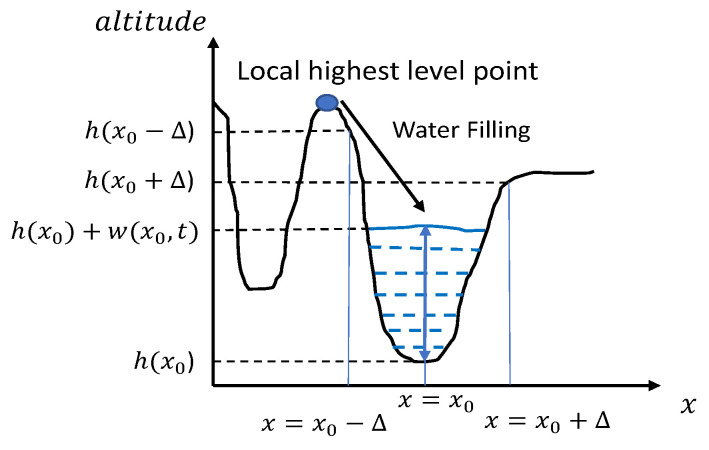
One-dimensional topographic model of a basin and its neighborhood, coupled with the water-filling direction.

We set h(x) as the altitude of the topographic surface and w(x,t) as the water level at a point of time *t*. For a point $x_{0}$, its overall altitude $K(x_{0},t)$ is the sum of $h(x_{0})$ and $w(x_{0},t)$, i.e., $K(x_{0},t)$ = $h(x_{0})$ + $w(x_{0},t)$. Figure 3 illustrates a one-dimensional model of plateau and basin. Specially, an essential constraint about w(x,t) is given as follows

(1) w(x,t)≥0,∀t,∀x∈I

where *I* is denoted as the domain of an image. To evaluate w(x,t), the inflow and outflow of water are modeled by three parts as below.

**Where does the “water” come from?** The water is simulated at the pixel-wise in the input image, which is similar with the techniques developed by [24,41,42]. In our study, locality means that the water comes from the neighboring pixels, in other words, the pixel with the highest intensity (or altitude) is selected as water source. It is denoted by

(2) $$h_{m}\left(x_{0}\right)=max\left\{h_{x}\right\},h_{x}\in NeighboringPixels$$

NeighboringPixels represents a number of neighboring pixels of point $x_{0}$. It can be concluded that $h_{m}\left(x_{0}\right)\geq h_{x}$. Thus, to meet Equation (1), the flooding process can be modeled by

(3) $$w_{f}\left(x_{0},t\right)=h_{m}\left(x_{0},t\right)-K\left(x_{0},t\right)$$

**Where does the “water” flow out?** We consider the effusion process through the pixel’s surroundings in a dynamic changing manner. The effusing process for 1D case can be modeled by

(4) $$\begin{array}{c} w_{e}\left(x_{0},t\right)\propto min\left\{K\left(x_{0}+\Delta ,t\right)-K\left(x_{0},t\right),0\right\}\\ +min\{K\left(x_{0}-\Delta ,t\right)-K\left(x_{0},t\right),0\} \end{array}$$

It can be seen that the $w_{e}\left(x_{0},t\right)$ is non-positive, which represents the amount of effusion water for point $x_{0}$. The water only flows into the lower places.

**How is the “water” stored?** The change in water level depends on flood and effusion results, and it is the sum of the two components. Meanwhile, considering the previous water level, the final altitude of $x_{0}$ is formulated by an iterative form

(5) $$K\left(x_{0},t+\Delta \right)=K\left(x_{0},t\right)+w_{f}\left(x_{0},t\right)+\alpha \cdot w_{e}\left(x_{0},t\right)$$

For a 2D image, the iterative update process of the overall altitude can be written as

(6) $$\begin{array}{cc} K\left(x_{0},y_{0},t+\Delta_{t}\right)=K\left(x_{0},y_{0},t\right)+\left(h_{m}\left(x_{0},y_{0},t\right)-K\left(x_{0},y_{0},t\right)\right)\; & \\ +\alpha \cdot \{min\left\{K\left(x_{0}+\Delta_{x},y_{0},t\right)-K\left(x_{0},y_{0},t\right),0\right\} & \\ +min\{K\left(x_{0}-\Delta_{x},y_{0},t\right)-K\left(x_{0},y_{0},t\right),0\}\; & \\ +min\{K\left(x_{0},y_{0}+\Delta_{y},t\right)-K\left(x_{0},y_{0},t\right),0\}\; & \\ +min\{K\left(x_{0},y_{0}-\Delta_{y},t\right)-K\left(x_{0},y_{0},t\right),0\}\} \end{array}$$

where $\Delta_{t}$ represents the changing time, $\Delta_{x}$ and $\Delta_{y}$ are defined as distances from $\left(x_{0},y_{0}\right)$ to its neighboring pixels. The α is an important parameter that controls the speed of the effusion process. α should be set carefully and it is expected to be limited in a suitable ratio in order to store the water. For LWF, the parameter α should be no greater than 0.25 due to the use of four neighboring points. In practice, α=0.22 may provide a satisfactory result. The iteration process will come to an end if the difference between two continuous altitudes is small enough or it reaches the maximum iteration number. Three iterations is enough to generate a proper shading map that represents the local background color. The shading map can be used to separate umbra and penumbra.

### 2.2. Separate Umbra and Penumbra

The shading map in Figure 2b is an image with three channels. To obtain the umbra and penumbra mask, a series of steps are designed to reach the goal.

Firstly, for each channel, a medium filtering and a binary threshold operation are adopted to generate a binary image, indicating shadow regions and unshadowed regions. Then, three channels are merged together. For a point, at least one of the three channels must be classified as shadow. It will be regarded as an umbra point. The umbra mask can be obtained by the pixel classification one by one.

Next, umbra masking is performed on a succession of dilation operations, generating an expanded shadow mask. In practice, two times of dilation are expected to be enough. Finally, the expanded shadow mask is subtracted by the umbra mask, producing the penumbra mask. In Figure 2c, the blue and red represent umbra and penumbra, respectively.

### 2.3. Umbra Enhancement

For umbra enhancement, an effective strategy to correct illumination is to relight umbra based on Retinex theory [43]. It requires the calculation of an enhancement scale that can be expressed as a ratio between a global reference background color and a local background color. Let *G* be the global reference background intensity, it can be expressed by

(7) $$G_{i}=\frac{1}{n}\sum L_{i}\left(x,y\right),\left(x,y\right)\in Unshadowed\;Region$$

where $i\in \left\{r,g,b\right\}$, *n* represents the number of pixels in an unshadowed region, L(x,y) is the local background in Figure 2b. *G* is the global background color with three channels.

Then, the enhancement scale can be easily obtained through the equation $\eta \left(x,y\right)=\frac{G}{L(x,y)}$. Hence, umbra can be enhanced by a multiplication of a pixel’s intensity and the enhancement scale η(x,y).

Penumbra are located between umbra and lighted regions, and are generally regarded as the shadow boundaries. The penumbra varies widely and makes it difficult to estimate the enhancement scale. In this paper, we put forward a solution to solve the problem in the next section.

### 2.4. Local Binarized Water-Filling

**Figure 4:**
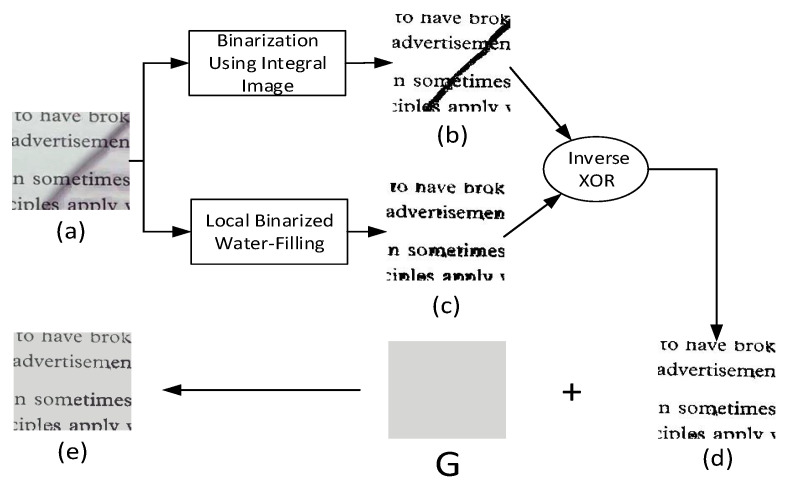
Shadow boundaries removal using the LBWF-based algorithm. (**a**) is the image from Figure 2b,d the binarization result of an adaptive thresholding method [20], (**c**) the binarization result of the proposed LBWF, (**d**) the inverse result of the XOR operation between the images (**b**) and the image (**c**), (**e**) the final result of shadow removal by combing the image (**d**) and the global background color *G*, which includes three channels and comes from Equation (7).

To solve the issue associated with the penumbra, we propose an algorithm to correct the illumination distortions, called the local binarized water-filling algorithm (LBWF-based algorithm). The overall structure of LBWF is similar to that of LWF, but there are some differences. Two main differences between LBWF and LWF are the following: the iteration number of LBWF is one; the parameter α of the effusion process is set to one. This setting of parameters not only speeds up the effusion process, but also reduces background noise. It is able to produce different and significant results compared with LWF. Experiments indicate that LBWF is more likely to suppress the effects of penumbra and keep the integrity of text, which can be found in Figure 4c.

LBWF is able to produce a gray-level image with only text and background, which is indicated in Figure 4c. The penumbra between text lines can be suppressed well, which verifies the effectiveness of LBWF. To obtain a better result, a binary image (Figure 4b) is generated by the integral image-based method [20]. Then, an inverse XOR operation is carried out to produce a clearer image. Finally, the global background color *G* is combined with Figure 4d to generate an unshadowed result (Figure 4e). Overall, the algorithmic description is presented in the form of pseudocode, as shown in (Algorithm 1).
**Algorithm 1** Algorithm of removing shadows from a document image.**Input:** A document image with shadows: **I**.**Output:** An unshadowed image: UI.1:Obtain the shading map **S** using local water-filling algorithm. Split image **I** into three channels in RGB color space. For each channel, Equation (6) is carried out in an iterative manner three times. Threshold parameter α=0.22. Merge the results of three channels into a shading map **S**.2:Separate umbra and penumbra. With **S** obtained, median filtering and OTSU binarization are operated for three channels, generating binary masks $BI_{B}$, $BI_{G}$, $BI_{R}$.A voting strategy of $BI_{B}$, $BI_{G}$, and $BI_{R}$ is used to determine the shadow region mask SR.SR:←ϕ(SR), ϕ represents the removal of the border noise with a size of 2 pixels.UmbraMask=ψ(SR), Umbra mask is generated by an dilation operation ψ on SR.DilatedUmbra←ψ(UmbraMask) is carried out at least two times.PenumbraMask=DilateUmbra−UmbraMask is to produce the penumbra mask.3:Umbra enhancement. Calculate the average background intensity in the non-shadow region by Equation (7): $G_{i}=\frac{1}{n}\sum L_{i}\left(x,y\right),\left(x,y\right)\in Unshadowed\;Region$.Compute umbra enhancement: $\eta \left(x,y\right)=\frac{G}{L(x,y)}$.Remove umbra: $UI_{umbra}\left(x,y\right)=I\left(x,y\right)\times \eta \left(x,y\right)$4:Penumbra removal using LBWF-based algorithm. Obtain a binarization image $B_{1}$ from $UI_{umbra}$ using integral image technique, which corresponds to Figure 4b.Compute a new binarization result $B_{2}$ from $UI_{umbra}$ using the Local Binarized Water-Filling technique. Split image $UI_{umbra}$ into three channels in the RGB color space. For each channel, Equation (6) are carried out once. Threshold parameter α=1.$B_{3}=InverseXOR\left(B_{1},B_{2}\right)$, this corresponds to Figure 4d.$UI\leftarrow B_{3}+G$5:**return**UI

## 3. Experimental Analysis

Visual and quantitative results are provided in this section. Our method runs on a PC with 3.5 GHz Xeon machine, and it is implemented by C++ and open source in computer vision (OpenCV) under the Visual Studio 2015 development environment. We compared our approach with two approaches whose codes are available online [11,24]. All the methods are performed on the same PC with a Windows 10 Operating System and 64GB RAM installed, and each method utilizes a suite of parameters. Each method runs five times to obtain the average running time.

### 3.1. Dataset

Previous researchers have proposed related datasets for shadow removal in document images, for example, the Adobe [14] and HS datasets [21]. To verify the proposed method’s effectiveness, these datasets are selected for the evaluation. Since there are a few strong shadow datasets available for optical shadow removal, we create one for evaluation, which is called the OSR dataset. It consists of two parts: the first part contains 237 images (controlled group, OSR_CG) with ground truth which are created under a control environment, and the other has 24 images (natural group,OSR_NG) without ground truth which are obtained from the Internet or captured under natural scenes.

The OSR_CG was created in a room. The documents were taken from books, newspapers, booklets, etc. They are typical documents. In the process of creating the dataset, two persons worked together. Firstly, the document was fixed on a desk, and a smart phone holder was adjusted to ensure our iPhone XR was well positioned to take photos. Then, one person created the source light using a lamp and remained still at all times. The other person created occlusions using objects such as hands and pens. Each time, the moving magnitude of occlusion was as small as possible. The clear images were captured first and then the images with shadows were captured. To align shadow images and clear images, the iPhone XR was not touched, and images were captured and controlled using an earphone wire. The documents, desk, and the smartphone were not touched and their positions were not changed throughout the process. These measures can guarantee the ground truth captured under uniform white illumination.

**Figure 5:**
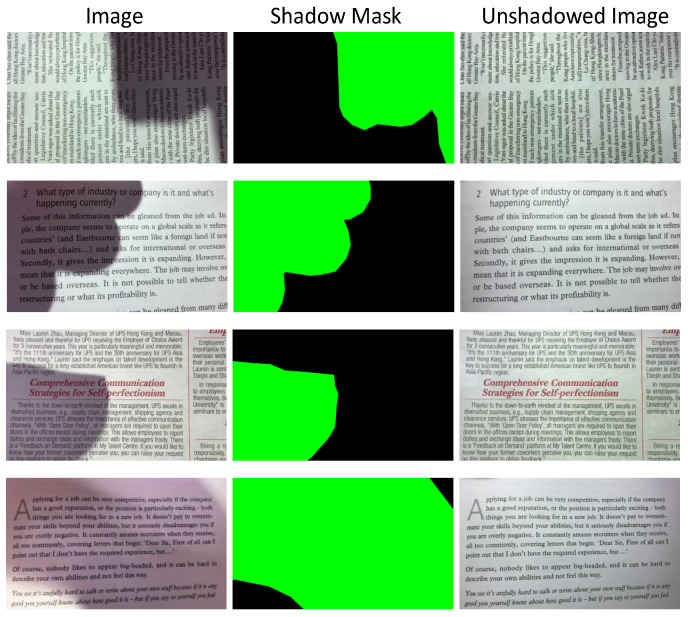
Some examples of the proposed dataset. Specifically, the middle column gives the shadow masks and the green areas indicate the shadow regions. The right column represents the ground truth.

The size of the controlled group is 960×544 (96 dpi), some examples are shown in Figure 5. We also built up the ground truth for shadow regions manually using photoshop, which can be employed for visual comparison and quantitative analysis. The images in the natural group are of different sizes and they are captured with various illuminations and shadow strengths. The OSR dataset is available to the public: “https://github.com/BingshuCV/DocumentShadowRemoval”.

### 3.2. Evaluation Metrics

To measure the effect of shadow removal, one of the most commonly used evaluation metrics is Mean Squared Error (MSE). It is defined by

(8) $$MSE\left(R,GT\right)=\frac{1}{n}\sum {\left(R(x,y)-GT(x,y)\right)}^{2}$$

where *R*, GT, and *I* represents the result image after shadow removal, ground truth, and input image, respectively. *n* is denoted as the number of pixels. This metric is widely used to evaluate the quality of algorithms. Further, we also employed an evaluation metric ErrorRatio [44] for the assessment of methods, which is shown as follows:

(9) $$ErrorRatio=\frac{RMSE(R,GT)}{RMSE(I,GT)}$$

where RMSE is the root MSE (i.e., $\sqrt{MSE}$). For an image, the area of shadow regions is usually uncertain. When the ratio of the shadow regions (i.e., the green parts labeled in the ground truth in Figure 5) to the whole image is small, the evaluation result may be influenced by the lighted regions (i.e., the black parts labeled in the ground truth in Figure 5). For fairness, only the shadow regions are considered in the evaluation.

In addition, the Structural SIMilarity (SSIM) index [45] is also considered for evaluating the structural similarity between the prediction and ground truth.

### 3.3. Comparisons with the State-of-the-Art Methods

**Figure 6:**
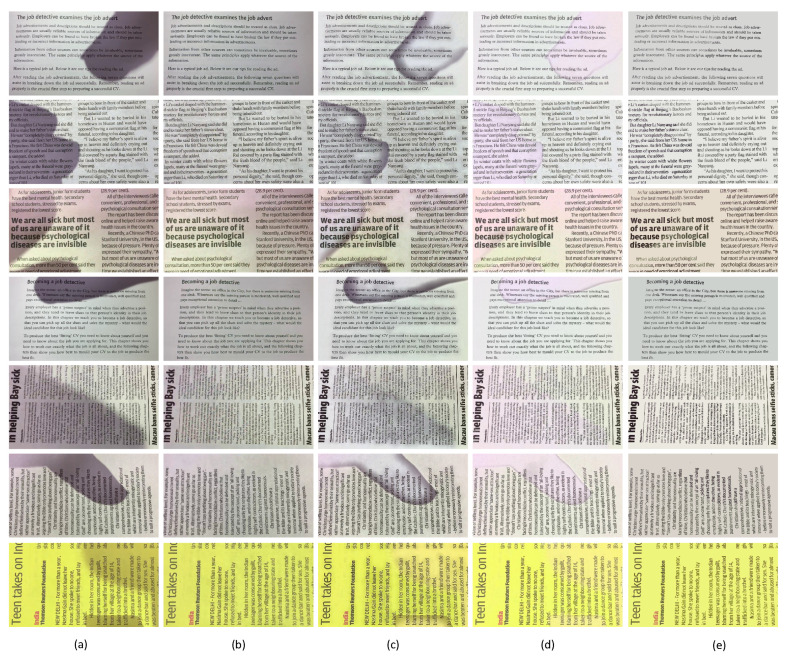
The visual comparisons of some state-of-the-art methods for the proposed OSR_CG dataset. (**a**) the input images, (**b**) the ground truth, (**c**) the results of [11], (**d**) the results of [24], (**e**) our results.

**Figure 7:**
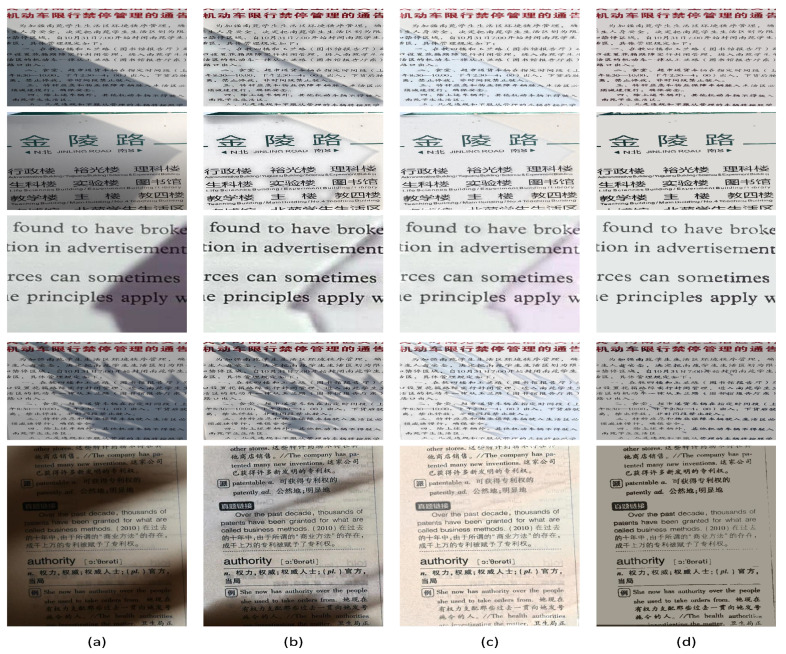
The visual comparisons of some state-of-the-art methods for the proposed OSR_NG dataset. (**a**) the input images, (**b**) the results of [11], (**c**) the results of [24], (**d**) our results.

**Figure 8:**
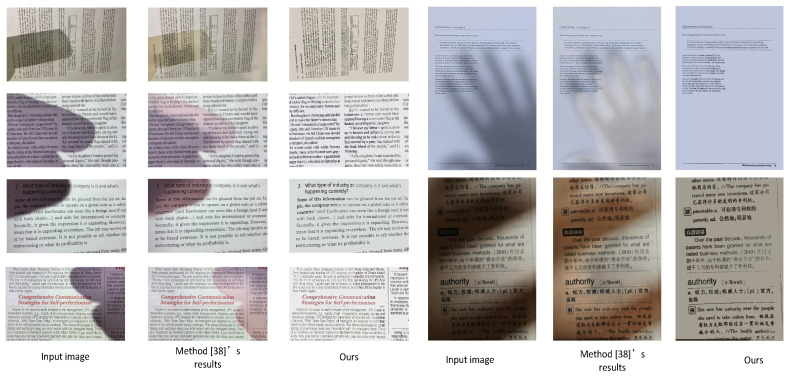
Visual comparison between our method and a CNN model [38].

In comparison to the state-of-the-art methods, we choose a water-filling method [24] and a 3D point cloud-based method [11]. Both represent state-of-the-art techniques for shadow removal in document images. Specifically, we compared these with a CNN model [38]. Quantitative comparisons are presented in Table 1, Table 2 and Table 3. Visual comparisons are shown in Figure 6, Figure 7 and Figure 8.

#### 3.3.1. Quantitative Comparison

In terms of quantitative comparisons, we utilize three evaluation metrics: the MSE,ErrorRatio,SSIM. For MSE and ErrorRatio evaluation metrics, lower values indicate that the method can remove shadows effectively and the produced images are closer to the ground truth. For SSIM, the higher the better.

It can be seen from Table 1, Table 2 and Table 3 that our results are much lower than those the methods in [11,24]. For example, in Table 1, our ErrorRatio is only 21.65% of method [11], 10.28% of method [24]; our MSE=105.8 is much lower than those of method [11] with MSE=2062.2, method [24] with MSE=9167.0. Meanwhile, Table 2 and Table 3 also demonstrate that our method is superior to the methods in [11,24].

The metric SSIM values of the methods are relatively close to each other, but there are differences. Table 1 shows that our method (0.927) is higher than the approach in [11] (0.802) and the approach in [24] (0.683). In Table 2, our method achieves 0.885, better than 0.878 of [11] and 0.861 of [24]. Although our method is inferior to the compared methods in Table 3, the differences are relatively small.

Therefore, our method performs better than the state-of-the-art methods [11,24] in the evaluation metrics. The performance differences are statistically significant. The advantages of the proposed method are demonstrated.

Moreover, we also provide the running time comparison by conducting methods on an image size of 960×544 pixels. Our method takes 0.265 s to process one frame, only accounting for one-sixth of the computational cost of the method [24]. A large number of water-filling processes designed in [24] can lead to an large increase of computational cost. As can be seen from the Table 3, the approach proposed by [11] requires 8.84 s to remove shadows for one frame, which is almost over 6 times the computational cost of [24] and 34 times the computational cost of our method. The reason why the method [11] runs slowly is because it spends a long time on visibility detection at the 3D point cloud transformation stage.

The computational complexity of [11] is O(nlogn) for *n* points. For [24], its computational complexity is O(pn) for *n* points, *p* is the number of iterations. Our method’s computational complexity is O(mn) for *n* points, *m* is the running time of local water-filling. *m* is less than *p*. Meanwhile, the number of iterations *p* or runs *m* is always set as a constant value and is far lower than the number of points *n*. Thus, the computational complexities of the method in [24] and ours are of a similar level and far fewer than that of the method in [11].

#### 3.3.2. Visual Results

It can be seen from Figure 6 that visual comparisons of seven images with shadows are presented. Our method achieves better visual results than the compared approaches.

The approach in [24] employed a global incremental filling of catchment basins and corrected illumination distortions on the luminance channel of the YCbCr color space. It is based on the assumption that the color information of shadow regions remains unchanged while the intensity decreases. Figure 6 shows that the method in [24] produces unnatural colors, for example, the shadow regions become pink. This is because for strong shadows, the assumption in [24] is hard to meet. The approach [11] produces many artifacts on shadow boundaries, making the image difficult to perceive visually. The reason may derive from the fact that the 3D point cloud transformation is not able to distinguish shadow points from texts due to the high similarity between some shadow points and text.

The proposed method is inspired by the techniques in [24,41,42] and implemented based on RGB color space, which is defined by the three chromaticities of the red, green, and blue. The method presents a new way to process umbra and penumbra, respectively. By integration with the LBWF-based module, shadow boundaries can be addressed appropriately. The color information belonging to shadow regions appears more natural.

To further demonstrate the effectiveness of our method, we conducted experiments on natural images shown in Figure 7. It can be seen from the figure that the approach in [11] has issues when dealing with nonuniform, strong shadows and the approach in [24] tends to change the color of output images. The proposed method may generate clean unshadowed images.

Figure 6 and Figure 7 show that the methods in [11,24] produce more artifacts than ours, which is in accordance with the quantitative comparison in Table 3. Visual comparisons and quantitative results demonstrate the effectiveness and efficiency of our proposed method.

#### 3.3.3. In Comparison with a Deep Learning Method

Convolutional Neural Network (CNN) models, as a representative of deep learning techniques, have achieved impressive results in various fields. Recently, some CNN models of shadow removal have been proposed to process natural images and these have performed well. To compare with other existing deep learning methods, herein, we compare with a CNN model proposed by [38]. The CNN model can only process an image size of 640×480. Therefore, the test images need to be adjusted to this size and then processed. The comparison results are presented in Figure 8. It is pretty clear that many artifacts are left using the approach in [38], resulting in an image that is difficult to percieve visually. The possible reason for this is that the approach in [38] was originally designed to remove shadows from natural images. Thus, it is not suitable for use on images of documents. One potential solution to this problem is to fine-tune a model on a document shadow dataset and redesign the CNN structure. In this regard, the training data should be prepared appropriately in future. In contrast, our method can remove shadows effectively.

**Figure 9:**
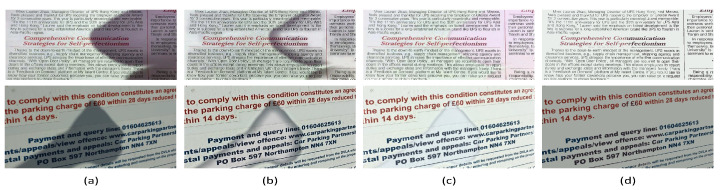
The visual comparisons of some state-of-the-art methods for text shadow removal. (**a**) the input images, (**b**) the results of [11], (**c**) the results of [24], (**d**) our results.

It should be noted that the results of some scenarios need to be improved, which is shown in Figure 9. When the colored text is covered with strong shadows, e.g., the red text in the first row and the blue text in the second row, the output text of our method tends to be black. The color degradation might lead to visual inconsistencies. Color constancy methods [26,27] could be considered to address this issue. In this regard, more research needs to be invested in the future.

## 4. Conclusions

In this paper, we proposed a local water-filling-based method for shadow removal. The main objective was to build up a topographic structure using pixels of a document image. An LWF algorithm was developed to estimate the shading map, which was used to divide shadows into umbra and penumbra. We adopted a divide-and-conquer strategy to process umbra and penumbra. Umbra was enhanced by Retinex theory, and penumbra was handled by the proposed LBWF-based algorithm. The strategy offers a powerful way to eliminate shadows, particularly strong shadow boundaries, and produce a clear and easy-to-read document. Moreover, a dataset was created that includes images with strong shadows and is available to the public. Experimental results performed on three datasets indicate that the proposed method outperforms some state-of-the-art methods in terms of effectiveness and efficiency.

Although our method is expected to be a promising technique for document binarization and recognition, we must to point out that the proposed method might produce unsatisfactory results when the shadow regions contain colored text. The output text tends to be dark and lack color information. It may bring discordant visual perception and this limitation will be addressed in the future work.

## Figures and Tables

**Table 1:** Quantitative comparisons of our method and some state-of-the-art approaches for the Adobe dataset with evaluation metrics MSE, ErrorRatio, SSIM.

Evaluation Metric	MSE	Error Ratio	SSIM
Kligler et al. [11]	2062.2	2.9489	0.802
Jung et al. [24]	9167.0	6.2104	0.683
Ours	105.8	0.6385	0.927

**Table 2:** Quantitative comparisons of our method and some state-of-the-art approaches for the HS dataset with evaluation metrics MSE, ErrorRatio, SSIM.

Evaluation Metric	MSE	Error Ratio	SSIM
Kligler et al. [11]	517.6	0.5641	0.878
Jung et al. [24]	1287.3	0.8980	0.861
Ours	158.2	0.3059	0.885

**Table 3:** Quantitative comparisons of our method and some state-of-the-art approaches for the OSR_CG dataset with three metrics: MSE, ErrorRatio, and the average running time on an image with a size of 960×544 pixels.

Evaluation Metric	MSE	Error Ratio	SSIM	Running Time (Seconds/Frame)
Kligler et al. [11]	1555.2	0.7160	0.892	8.84
Jung et al. [24]	2313.8	0.9216	0.885	1.396
Ours	1282.4	0.685	0.875	0.265
